# On the interpretation of annual oscillations in ^32^Si and ^36^Cl decay rate measurements

**DOI:** 10.1038/s41598-021-95600-8

**Published:** 2021-08-06

**Authors:** S. Pommé, K. Pelczar, K. Kossert, I. Kajan

**Affiliations:** 1 European Commission, Joint Research Centre (JRC), Geel, Belgium; 2 Physikalisch-Technische Bundesanstalt (PTB), Braunschweig, Germany; 3 Paul Scherrer Institut (PSI), Villigen, Switzerland

**Keywords:** Nuclear physics, Astronomy and astrophysics, Solar physics

## Abstract

The ^32^Si decay rate measurement data of Alburger et al. obtained in 1982–1986 at Brookhaven National Laboratory have been presented repeatedly as evidence for solar neutrino-induced beta decay. The count rates show an annual sinusoidal oscillation of about 0.1% amplitude and maximum at February–March. Several authors have claimed that the annual oscillations could not be explained by environmental influences on the set-up, and they questioned the invariability of the decay constant. They hypothesised a correlation with changes in the solar neutrino flux due to annual variations in the Earth-Sun distance, in spite of an obvious mismatch in amplitude and phase. In this work, environmental conditions at the time of the experiment are presented. The ^32^Si decay rate measurements appear to be inversely correlated with the dew point in a nearby weather station. Susceptibility of the detection set-up to local temperature and humidity conditions is a likely cause of the observed instabilities in the measured decay rates. Similar conclusions apply to ^36^Cl decay rates measured at Ohio State University in 2005–2012.

## Introduction

There is a rich bibliography from authors who have questioned the invariability of nuclear decay constants and the ensuing validity of the exponential-decay law^1^. Contrary to the notion of radioactive decay as a spontaneous process, new hypotheses were put forward in which solar neutrinos, cosmic neutrinos, gravitation waves, cosmic weather, etc. are actors influencing the radioactive decay process. Most of these claims of new physics have been convincingly invalidated by high-quality experiments showing invariability of the decay constants^2–22^, such that the discussion is largely settled. Nevertheless, some authors^1,23–28^ keep bringing up the case of the ^32^Si (and ^36^Cl) decay rate measurements by Alburger et al.^29^ as an argument in favour of neutrino-induced beta decay. The experimenters reported that “*small periodic annual deviations of the data points from an exponential decay were observed, but were of uncertain origin*”.

**Figure 1:**
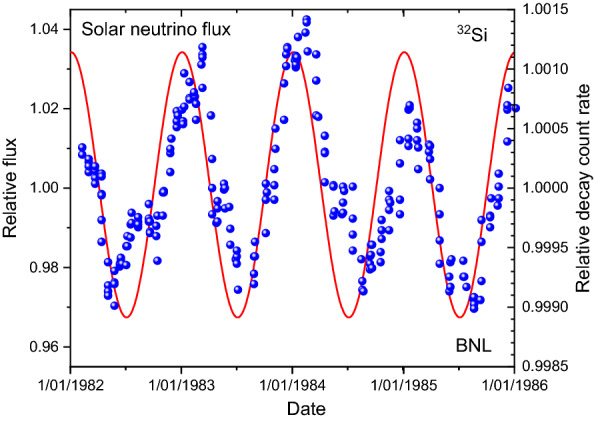
The relative variations in the ^32^Si decay rates from BNL (dots) relative to the variations of the solar neutrino flux impinging on Earth along its elliptic track around the Sun (line).

The often-repeated argument is that the annual oscillations in the decay rates over the period 1982–1985 cannot be explained by environmental conditions, such as ambient temperature and pressure. As an alternative explanation, some authors speculate that the cyclic deviations from exponential decay reveal decays induced by solar (or cosmic) neutrinos. The solar neutrino flux is inversely proportional to the Earth-Sun distance, which inherently leads to an annual oscillation of 3.3% amplitude with a maximum around 3 January. The relative deviations of the ^32^Si decay rates, however, have an amplitude of 0.3% and a maximum near 1 March. As demonstrated in Fig. 1, there is no match between both cycles, neither in amplitude nor in phase. There is a weak correlation (*R*^2^ = 0.236) between the expected neutrino flux and the measured decay count rates.

**Figure 2:**
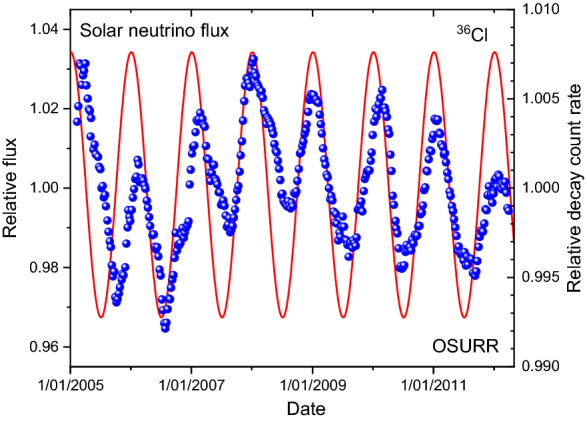
The relative variations in the ^36^Cl decay rates from OSURR (dots) relative to the variations of the solar neutrino flux impinging on Earth along its elliptic track around the Sun (line).

In the literature, there are additional data sets of repeated decay rate measurements of beta emitters^1,24^. An interesting example is the ^36^Cl decay rate series measured at the Ohio State University Research Reactor (OSURR) from 2005 to 2012, shown in Fig. 2. Annual oscillations of about 0.5% amplitude are apparent in this data set, as well as an additional slow instability component spanning a multi-annual period. Whereas the deviations from exponential decay sometimes overlap with extrema of the solar neutrino flux, most of the periods are not exactly in phase.

In this paper, the statement in various publications^1,23–28^ that *the annual oscillations in the *^*32*^*Si and *^*36*^*Cl decay rates cannot be ascribed to environmental parameters* is scrutinised. Historical meteorological data have been downloaded from weather stations in the neighbourhood of the laboratories at which the experiments were run. In particular, a possible causal relationship with temperature and humidity is investigated.

## ^32^Si

### Experiment

**Figure 3:**
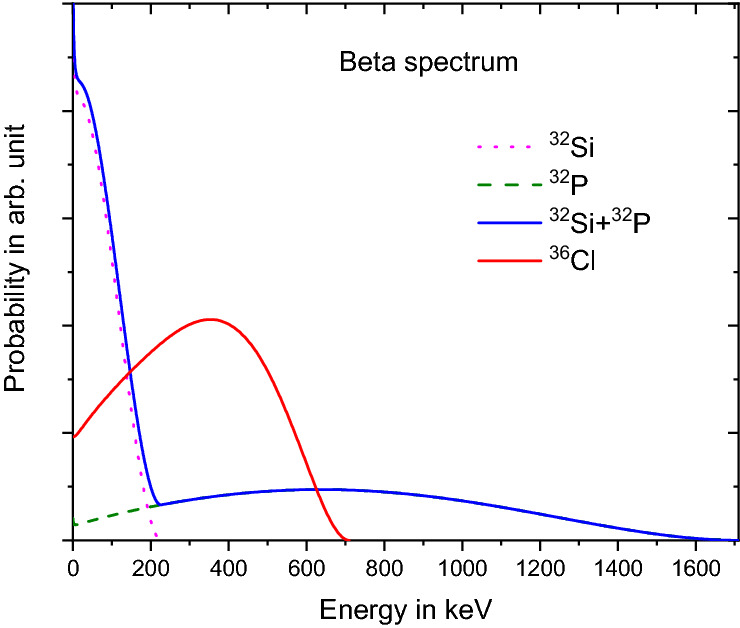
Beta emission spectrum of ^32^Si, ^32^P, ^32^Si + ^32^P in secular equilibrium, and ^36^Cl calculated from theory^34^. The measured spectra in the gas detector are degraded by energy loss and absorption of electrons in materials.

The decay rate of a ^32^Si beta-emitting source was measured with an end-window gas-flow proportional counter system at the Brookhaven National Laboratory (BNL) in the frame of a half-life determination^29^. The ^32^Si was measured in secular equilibrium with its progeny ^32^P. Using an automatic precision sample changer, the measurements were interwoven with decay-rate measurements of a long-lived ^36^Cl check source, to compensate for instabilities of the instrumentation. The radionuclides ^32^Si^30^ and ^32^P^31^ mainly decay through beta transitions with an allowed nature, whereas ^36^Cl^32^ decays predominantly (98.1 (1)%) via a second-forbidden non-unique beta transition and a weak (1.9 (1)%) electron-capture branch. The maximum beta energies^33^ amount to 227.2 (3) keV (^32^Si), 1710.66 (21) keV (^32^P), and 709.55 (5) keV (^36^Cl), respectively. The energy spectra were calculated with the BetaShape software^34^ and are shown for comparison in Fig. 3. The actual energy distribution of beta particles leaving the ^32^Si/^32^P source is smeared out due to the effective source thickness of 17 mg cm^−2^^29^, such that a significant fraction of the low-energy electrons will be stopped in the source, air, or entrance window before reaching the sensitive detector volume. The fraction of undetected electrons depends on the energy loss and threshold setting. The low-energy electrons (< 2.5 keV) and few X rays emitted after electron-capture decay of ^36^Cl should not play a significant role.

Alburger et al.^29^ discuss at length the measurement conditions that could influence the stability of the experiment: “Since the β rays pass through several millimetres of air before entering the counter, the entire apparatus is enclosed in a pressure-regulated box in order to minimize differential energy-loss effects that would occur with changes in barometric pressure. […] The various factors that can affect the counting rate include the voltage on the counter, the rate of gas flow in the counter, the amplifier gain and discriminator setting, the box pressure, air temperature, relative humidity, and source positioning. […] Changes in both temperature and relative humidity will result in changes of air density, assuming the box pressure is held constant, and these density changes can be translated into equivalent pressure changes. A change in temperature of 2.0°F would result in a one standard deviation change in the ^32^Si/^36^C1 ratio. A change in relative humidity from 30 to 80% would also cause a variation of one standard deviation.”

### Temperature and humidity

In an advanced stage of the experiment, Alburger et al.^29^ noticed that variations in the count rates showed ‘a curious correspondence’ with the curve of daily average temperature in the New York area. They regretted that “the chart recording of temperature and relative humidity in the room containing the counting system was carried out only during the final 5 months of the experiment, after it was realized that variations in these parameters might be responsible for the observed fluctuations. Average values of the temperature measured during these runs were in the range 72.4–74.7°F and the average relative humidity varied from 35 to 76%. From a study of records available for other areas in the Chemistry building, it seems likely that the average temperature in our room during the entire course of the experiment remained well within the range 70–76°F. Thus, of all the parameters either tested or considered, temperature and relative humidity seem to be the only ones that could affect the ^32^Si/^36^C1 ratio at the level of one standard deviation.”

**Figure 4:**
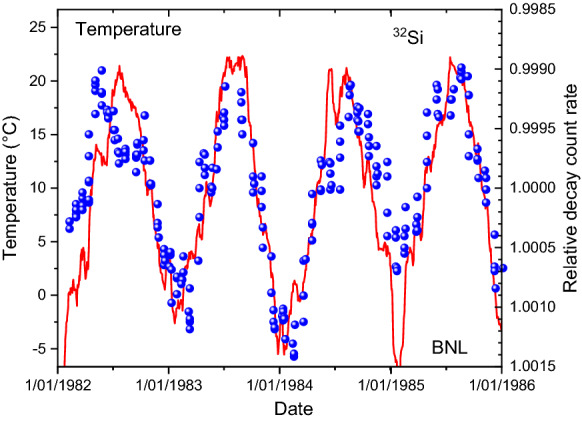
Relative deviations on measured decay rates of ^32^Si at BNL (dots), compared to temperature data from a nearby weather station (line).

**Figure 5:**
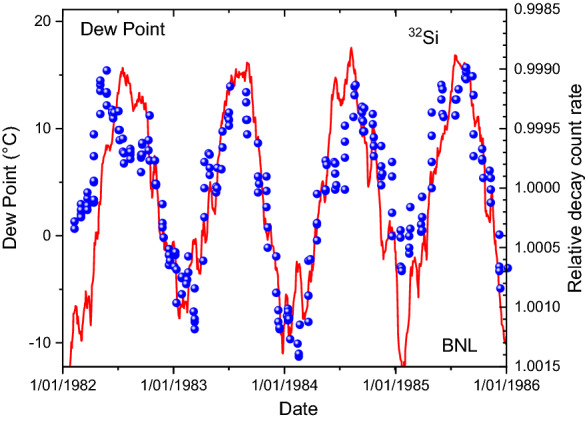
Relative deviations on measured decay rates of ^32^Si at BNL (dots), compared to dew point data from a nearby weather station (line).

**Figure 6:**
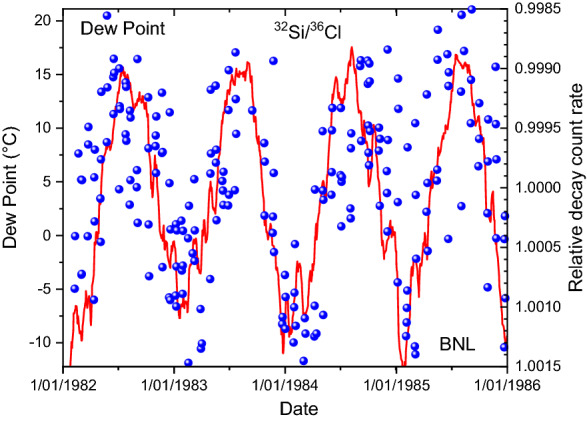
Relative deviations on measured decay rate ratios of ^32^Si/^36^Cl at BNL (dots), compared to dew point data from a nearby weather station (line).

Nowadays, meteorological data at the time of the experiment can be downloaded from websites of weather stations. Historical data of temperature, dew point, humidity, wind speed, and air pressure are available from the station at Ronkonkoma (NY, USA)^35^, situated at 22 km distance from Upton, the home town of the Brookhaven National Laboratory. In Fig. 4, the variation in the measured ^32^Si decay rates are plotted together with a moving average of the temperature values at Ronkonkoma. Whereas both curve shapes do not match to perfection, there is an obvious anti-correlation between temperature and count rates in the beta counter (*R*^2^ = 0.376). The match between dew point and count rate instabilities, shown in Fig. 5, appears to be marginally better (*R*^2^ = 0.381). The outside weather conditions are likely to have influenced the decay rate measurements. Taking the ratio of measured ^32^Si/^36^C1 decay rates, presented in Fig. 6, does not fully compensate for the annual effect.

### Interpretation

Since the electrons enter the detector with a continuous energy spectrum, it is the threshold level that separates the detected from the non-detected fraction. Any change in the threshold energy, the amplification of the gas detector, or the energy spectrum of the electrons (after straggling in air with a variable density) will result in a different effective detection efficiency. The assertion by Alburger et al. that temperature and humidity are the most likely factors to have influenced the measurement seem justified from Figs. 4, 5 and 6. Surely, the agreement in temporal behaviour is more convincing than with the solar neutrino hypothesis depicted in Fig. 1. It was mentioned in the original paper that the gas pressure regulation in the detector failed sometimes, in particular at low barometer due to insufficient pressure differential with the reference pressure. This artefact may have contributed to a partial mismatch between decay rates and dew point.

## ^36^Cl

### Experiment

The experimental details of the ^36^Cl decay rates collected at the OSURR in Columbus, Ohio (USA) have been described by Jenkins et al.^24^. Over the course of 7 years, the ^36^Cl source was measured weekly as part of the efficiency check of the Eberline Beta Counter BC-4 that was used for counting contamination survey wipes. The detector consisted of a 4.4 cm diameter pancake-style Geiger–Müller tube, which was protected with 2.22 cm of lead against background radiation. The check source consisted of two aluminium half-disks, each having machined circular depressions to hold the activity. After early 2006, a more reproducible source geometry inside the detector was assured by centring the source on the tray by means of an aluminium disk insert. Typical ^36^Cl count rates were 1038 s^−1^, with a relative uncertainty of 0.28%. Typical background count rates were approximately 0.35 s^−1^, with a relative uncertainty of 6.7%.

### Temperature and humidity

**Figure 7:**
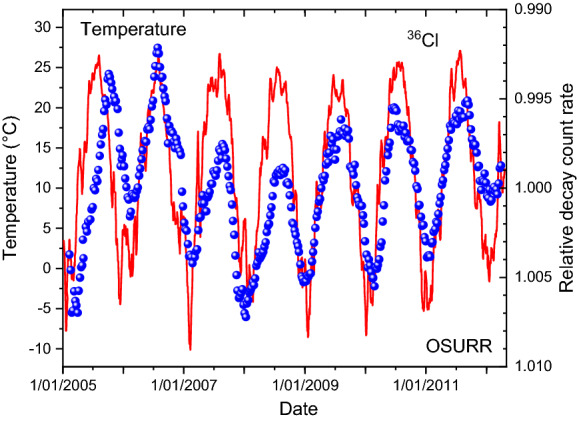
Relative deviations on measured decay rates of ^36^Cl at OSURR (dots), compared to temperature data from a nearby weather station (line).

**Figure 8:**
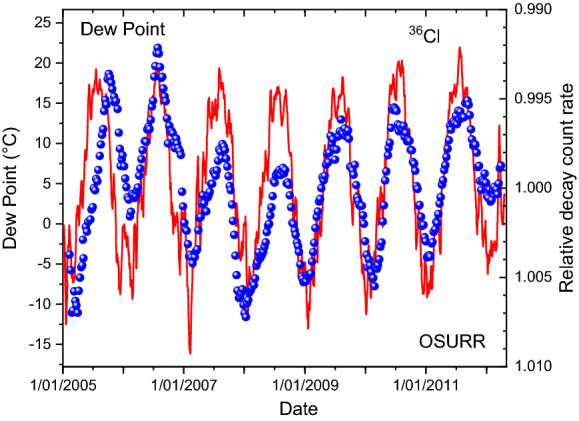
Relative deviations on measured decay rates of ^36^Cl at OSURR (dots), compared to dew point data from a nearby weather station (line).

**Figure 9:**
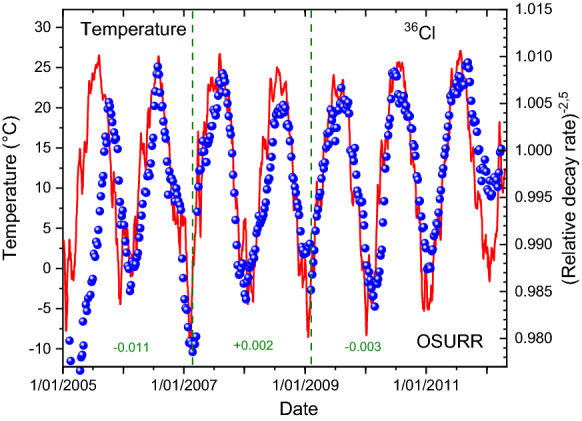
Local shift and power of − 2.5 applied to relative deviations on measured decay rates of ^36^Cl at OSURR (dots), compared to temperature data from a nearby weather station (line).

**Figure 10:**
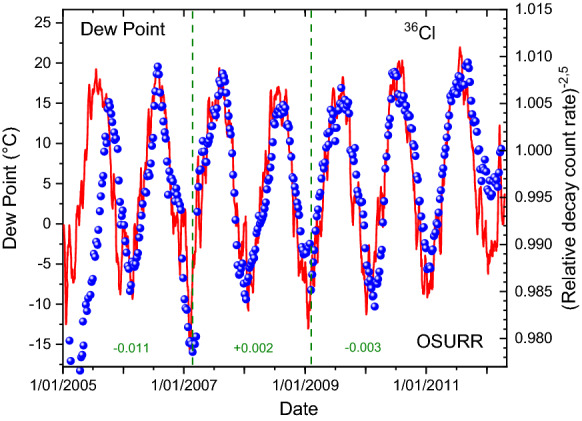
Local shift and power of − 2.5 applied to relative deviations on measured decay rates of ^36^Cl at OSURR (dots), compared to dew point data from a nearby weather station (line).

From the same meteorological service provider as for the case of ^32^Si, historical weather data were extracted for the International airport station at Columbus (OH, USA)^36^. In Figs. 7 and 8, the variations in the measured ^36^Cl decay rates are compared qualitatively with the temperature and dew point recordings, respectively. Except for the first year, there appears to be a very good correlation in time of the annual decay rate variations with weather changes, i.e. much better than with the solar neutrino flux in Fig. 2. However, there is a long-term drift in the ^36^Cl data set which complicates a more direct comparison of the peak shapes. This was remediated in Figs. 9 and 10 by means of a piecewise analysis in which the ^36^Cl data were shifted independently in three time zones spanning 2, 2, and 3 years respectively, and a power of -2.5 was applied to the relative fluctuations. There is a very strong correlation (*R*^2^ = 0.80) of the shifted decay data from the period 2006–2011 and the associated average dew point or temperature values.

### Interpretation

The strong correlation between dew point and decay rate instabilities (shifted and to the power of − 2.5) in Fig. 10 is extraordinarily convincing. The data in 2005 seem shifted downwards, possibly due to a difference in counting geometry, since the aluminium centring piece was introduced in early 2006^24^. There is also a moderate shift at the end, in 2012, which remains unexplained. The overall excellent match between activity and weather data underlines the impact of humidity and temperature on the relative detection efficiency in the G–M counter. In recent research, the importance of humidity on a G–M counter has been demonstrated quite convincingly through a simple humidity accumulation model, in a case where decay rate changes had been erroneously associated with space weather^21,22^.

The question arises why some authors^1,23–28^ presume the stability and robustness of G–M systems, whereas an unbiased investigation should have made clear that these instruments are sensitive to temperature and humidity. Anybody could have reached this conclusion by using the publicly available weather data. The lack of critical scrutiny by authors defending the ‘neutrino-induced decay’ hypothesis bears the hallmark of cognitive bias. The problem lies not in the liberty taken to suggest new ideas, but in the lack of scrutiny to put these ideas to the test. In the words of Carl Sagan ^37^: “At the heart of science is an essential balance between two seemingly contradictory attitudes—an openness to new ideas, no matter how bizarre or counterintuitive they may be, and the most ruthlessly sceptical scrutiny of all ideas, old and new. This is how deep truths are winnowed from deep nonsense.”

## Conclusions

There is a strong correlation between the variations of the decay rates of ^32^Si measured at BNL and of ^36^Cl at OSURR with the temperature and dew point outside the laboratories where they were measured. Influences of temperature and humidity on air density (energy straggling of electrons) as well as gas detectors and their electronics (threshold and amplification) are plausible causes for instability in the decay rate measurements. There is little convincing correlation with the expected annual changes in the solar neutrino flux impinging on Earth. Consequently, there is no reason to infer that these often-referenced cases support in any way the unfounded conjecture of neutrino-induced beta decay.

## Data Availability

The data that support the findings of this study are weather data publicly available on the internet, and decay rate measurement data not publicly available, being in the hands of third parties.
